# Chikungunya and Zika Viruses: Global Emerging Health Threats

**DOI:** 10.4269/ajtmh.18-0613

**Published:** 2018-10

**Authors:** Bradley J. Blitvich, Aaron C. Brault

**Affiliations:** Department of Veterinary Microbiology and Preventive Medicine College of Veterinary Medicine Iowa State University Ames, Iowa E-mail: blitvich@iastate.edu; Division of Vector-Borne Diseases Centers for Disease Control and Prevention Fort Collins, Colorado E-mail: zlu5@cdc.gov

Chikungunya virus (CHIKV; family Togaviridae) and Zika virus (ZIKV; family Flaviviridae) are mosquito-transmitted viruses that were previously confined to Africa and Asia and occasionally associated with localized outbreaks and sporadic cases of mostly non–life-threatening disease in humans; thus, both viruses have historically received limited attention from the research community. However, in recent years, CHIKV and ZIKV have caused explosive outbreaks in numerous countries in the Eastern and Western Hemispheres, placing both viruses into the international spotlight. Evidence is accumulating that CHIKV infections can progress to fatal encephalitis, and recent ZIKV outbreaks have been accompanied by Guillain–Barré syndrome and microcephaly, two devastating conditions rarely if ever previously associated with arthropod-borne virus (arbovirus) infections. Consequently, these two previously neglected viruses are now the focus of high-priority research, have received widespread media coverage, and are known to the general public.

*Chikungunya and Zika viruses: Global Emerging Health Threats* (2018; Academic Press, an imprint of Elsevier) provides a comprehensive and engrossing review of CHIKV and ZIKV. The decision to review CHIKV and ZIKV in the same book is logical; both viruses are maintained in similar transmission cycles, originated in Africa, and appear to have invaded the Western Hemisphere in the same year. The first outbreak of CHIKV in the Western Hemisphere occurred on the Caribbean Island of Saint Martin in 2013. Zika virus was detected in Brazil in 2015, but molecular clock studies have provided evidence that the virus was circulating undetected as early as 2013. The book was edited by three highly accomplished and well-respected arbovirologists: Stephen Higgs and Dana L. Vanlandingham, both from Kansas State University, and Ann M. Powers from the Centers for Disease Control and Prevention. The book contains 12 chapters, with contributions from an impressive ensemble of internationally recognized scientists.

The first chapter, written by Powers, provides a compelling overview of the pioneering work that led to the discoveries of CHIKV and ZIKV. The clinical features associated with the first documented outbreaks and the early field and laboratory studies performed in response to these outbreaks are discussed. The second chapter, written by Higgs and Vanlandingham, provides detailed information on the transmission cycles of CHIKV and ZIKV. The authors describe the virus, vector, and host interactions that drive transmission, and the barriers within the mosquito that the virus must overcome for transmission to occur. The chapter also lists potential vector spp. of each virus according to geographic region. Alternate modes of transmission (i.e., vertical transmission) are also discussed. Chapter 3 (by Halstead) provides an excellent review of the clinical manifestations associated with CHIKV and ZIKV infections. The author first describes the symptoms, treatment, and prognosis of patients with chikungunya. Zika syndrome is described next, with specific sections on febrile exanthem, Guillain–Barré syndrome, and congenital Zika syndrome. The chapter concludes with a review of the management and prognosis of patients with ZIKV syndrome.

Chapter 4 (by Diallo and others) provides a comprehensive account of the emergence of CHIKV and ZIKV in Africa, which is where both viruses were originally isolated and described. The authors did a remarkable job retrieving and compiling historical data. Multiple maps are provided to illustrate the geographic distributions of each virus in Africa, with data separated by laboratory technique and whether human, vertebrate animal, or mosquito investigations were performed. Detailed information on the vector and host ranges of each virus and the outcomes of vector competence studies are also provided. Chapter 5 (by Sam) is an outstanding review of CHIKV and ZIKV in Asia, defined as the region commonly referred to as Asia, in addition to Oceania and the Middle East. The chapter explains that the Asian and East/Central/South African (ECSA) genotypes of CHIKV were first detected in Asia in 1958 and 2005, respectively, although chikungunya-like outbreaks occurred as early as the 1700s. The introduction of the ECSA genotype resulted in an explosive outbreak that affected an estimated 1.38 million people in India in a few months alone. The emergence of ZIKV is also discussed, beginning with the initial outbreak that occurred on Yap Island in 2007.

Chapter 6 (by Zannoli and others) reviews the epidemiology and vector distribution of CHIKV and ZIKV in Europe. The chapter explains that CHIKV and ZIKV are not endemic in Europe because *Aedes aegypti* and *Aedes albopictus*, the principal vector spp., are not common. All reported autochthonous cases of CHIKV have been confined to Italy and France. There have been no reports of vector-borne transmission of ZIKV, although imported cases have been reported, and a few autochthonous cases have occurred due to sexual contact with infected travelers. Chapter 7 (by Vasconcelos and others) provides an intriguing overview of the recent introductions of CHIKV and ZIKV in the Americas and the explosive outbreaks that followed. The authors place an emphasis on the uncharacteristic features of the outbreaks: the rapid spread of both viruses, the unprecedented numbers of CHIKV and ZIKV cases, the unexpectedly high numbers of CHIKV- and ZIKV-associated fatalities, new mechanisms of ZIKV transmission (i.e., sexual and congenital transmission and blood transfusion), and association between ZIKV and pregnancy complications (microcephaly, miscarriage, and stillbirths). Detailed information is provided on the numbers of microcephaly cases reported in Brazil, the epicenter of the ZIKV outbreak.

Chapter 8 (by Huang and others) provides a well-written review of the genetics, molecular biology, and evolution of CHIKV and ZIKV. The genomic organization and the transcription and translation strategies of each virus are discussed. Crystal structures of viral proteins are also presented. In addition, genetic mutations associated with emergence are reviewed, including the E1 A226V variant of CHIKV that significantly enhances the infectivity of the ECSA genotype in *Ae. albopictus*. Chapter 9 (by Zannoli and others) provides an important review of the diagnostic and laboratory techniques available for each virus. The clinical, epidemiological, and laboratory criteria for the diagnosis of CHIKV and ZIKV infections are described. The types of specimens that are collected and laboratory techniques that are performed for chikungunya and Zika diagnosis are also reviewed. Laboratory techniques covered in the chapter include reverse transcription–polymerase chain reaction, virus isolation in cell culture, enzyme-linked immunosorbent assay, plaque reduction neutralization test, and immunofluorescence assay.

Chapter 10 (by Morrison) elegantly reviews the progress made in the development and characterization of animal models of CHIKV and ZIKV disease. The chapter describes animal models of CHIKV acute musculoskeletal disease, CHIKV chronic musculoskeletal disease, CHIKV encephalitis and other severe outcomes, ZIKV infection in adults and neonates, ZIKV sexual transmission, and ZIKV infection during pregnancy. Most work to date has been performed using rodents and nonhuman primates, although select other animals have also been evaluated. Chapter 11 (by Metz and Pijlman) provides an informative review of the progress made in the development of vaccines for CHIKV and ZIKV. Vaccine candidates based on several platforms are described (inactivated virus vaccines, live-attenuated virus vaccines, chimeric virus and viral vector vaccines, nucleic acid vaccines, and subunit and virus-like particle vaccines). As explained by the authors, four vaccine candidates for CHIKV have been tested in phase I clinical trials, including one that has entered phase II clinical trials. Zika virus vaccine development is more challenging because cross-reactive, non-neutralizing antibodies to a ZIKV vaccine could adversely affect dengue virus infected individuals by immune enhancement.

Chapter 12 (by Monath) provides an insightful prediction of future trends in CHIKV and ZIKV emergence, evolution, and transmission, in addition to their long-term impact on public health. It is predicted that the number of ZIKV cases in the Americas will decrease rapidly over the next 3 years, after which there will be low-level endemic transmission for 10 years, followed by a large epidemic. A similar cyclical pattern is suggested for CHIKV. The chapter includes a discussion on future research directions, most of which is devoted to vaccine and antiviral drug development.

To conclude, *Chikungunya and Zika viruses: Global Emerging Health Threats* is an excellent book. The editors assembled an impressive team of contributors, every chapter is informative and well written, and all major topics are covered. The book is a “must read” for all scientists and public health personnel with an interest in virology.

